# Computed Tomography (CT)-Based Morphometric Analysis of the Glenoid Fossa and Its Clinical Significance in the Lucknow Region, Uttar Pradesh

**DOI:** 10.7759/cureus.109492

**Published:** 2026-05-23

**Authors:** Akriti Anand, Garima Sehgal, Archana Rani, Anam Ahmad, Arvind K Pankaj, Rintu Biswas

**Affiliations:** 1 Anatomy, Hind Institute of Medical Sciences, Barabanki, IND; 2 Anatomy, King George's Medical University, Lucknow, IND; 3 Anatomy, Eras Lucknow Medical College and Hospital, Lucknow, IND

**Keywords:** glenoid cavity index, glenoid height, glenoid version, glenoid width, shoulder arthroplasty

## Abstract

Background and objective

The glenoid fossa is the lateral extension of the scapula's lateral angle, with a concave surface that articulates with the head of the humerus. Glenoid bone loss is frequently associated with glenohumeral instability. A thorough understanding of shoulder joint anatomy is essential, and the glenoid cavity index (GCI) provides a valuable measure for assessing the configuration and dimensions of the glenoid cavity. Restoring its normal anatomy improves joint function and reduces pain. Conventional two-dimensional (2D) and three-dimensional (3D) CT imaging remain the standard for evaluating the glenoid fossa and are crucial for preoperative planning in shoulder arthroplasty. This study was conducted to examine the morphometry of the glenoid fossa using CT imaging.

Materials and methods

CT scan data from 150 shoulder joints of 75 individuals (aged 30-70 years) were analyzed; the cohort included 35 males and 40 females. Scans were conducted using a Philips Brilliance 128-slice CT scanner at the Department of Radiodiagnosis, King George’s Medical University, Lucknow, Uttar Pradesh. This retrospective observational cross-sectional study included individuals who had undergone head and neck CT scans that clearly captured the shoulder joint region without any evident pathology. Glenoid width and height were measured using 3D reconstructed images, and the GCI was calculated as (width/height) × 100. Glenoid version (angle β) was assessed on 2D multiplanar images and recorded as positive for anteversion and negative for retroversion. The formula applied was $&beta; = &delta; &minus; 90&deg;$.

Results

The mean glenoid width and height on the left side were 23.04 ± 2.75 mm and 38.35 ± 4.26 mm, respectively; on the right side, they were 23.10 ± 2.45 mm and 38.14 ± 3.86 mm. The mean GCI demonstrated a statistically significant difference between males and females on the right side (p = 0.0159), whereas the difference on the left side was not significant (p = 0.064).

Conclusions

This CT-based study provides normative data on glenoid fossa dimensions, providing a valuable reference for surgeons and radiologists. Both sex and age should be taken into account during preoperative planning, as they are independent predictors of glenoid size, version, and index.

## Introduction

The glenoid fossa, positioned at the lateral angle of the scapula, is a shallow concave articular surface that articulates with the head of the humerus. The supraglenoid and infraglenoid tubercles, located at its superior and inferior margins, respectively, serve as attachment points for the tendons of the long head of the biceps brachii and triceps brachii [1]. The glenoid fossa is normally oriented with slight physiological anteversion or retroversion relative to the scapular body axis, with approximately 0 degrees considered the neutral glenoid version [2].

Variation in glenoid version and glenoid bone loss may affect glenohumeral joint stability and load distribution, contributing to instability, degenerative arthritis, and abnormal wear patterns. Therefore, precise assessment of glenoid orientation is essential for the evaluation of shoulder pathology and for preoperative planning of reconstructive shoulder procedures [2,3]. Burkhart and De Beer (2000) reported that shoulder instability recurrence rates after arthroscopic Bankart repair were significantly higher (67%) in patients with substantial glenoid bone loss, compared to 4% in patients without it. They coined the term "inverted pear glenoid" to describe cases in which over 25% of the glenoid width was lost [4]. 

The glenoid cavity index (GCI), calculated from glenoid height and width, helps determine glenoid configuration and anatomical variants. These metrics are crucial for diagnosing glenoid dysplasia, assessing the severity of retroversion, and selecting appropriate implant sizes in total shoulder arthroplasty (TSA) [5,6]. The GCI directly affects the biomechanics of the shoulder, guides surgical planning and prosthesis selection, and influences the long-term success of rotator cuff repairs [7,8]. Understanding the GCI also aids rehabilitation strategies and corrective procedures for glenoid deformities [7].

In reverse total shoulder arthroplasty (RTSA), accurate assessment of glenoid version and bone loss is essential for improving outcomes [9]. Farron et al. (2006) demonstrated that excessive retroversion (greater than 10°) during graft placement can result in graft loosening and micromotion at the bone-cement interface [10]. Two-dimensional (2D) axial CT scans are more precise than conventional radiographs in estimating glenoid version, making them the standard for preoperative assessment [11,12]. Studies also report that males generally exhibit greater glenoid retroversion than females [13]. Despite a typically neutral or mildly retroverted orientation in most patients, considerable individual variations must be taken into account [13,14]. CT imaging offers superior visualization of cortical and trabecular bone compared to MRI and enables multiplanar and 3D reconstructions, which are essential tools for preoperative planning. Therefore, this study was conducted to evaluate the morphometry of the glenoid fossa using CT imaging.

## Materials and methods

A total of 150 shoulder joint CT scan datasets from 75 individuals (age range: 30-70 years) were analyzed. Among the 75 participants, 35 were males, and 40 were females. These individuals had undergone head and neck CT scans that incidentally captured normal shoulder joints on either side. The study was conducted collaboratively by the Department of Anatomy and the Department of Radiodiagnosis at King George’s Medical University, Lucknow, Uttar Pradesh, India. Ethical approval was obtained from the Institutional Ethics Committee (Ref. No.: IV PGTSC-IIA/P36).

This retrospective observational cross-sectional study included individuals who had undergone head and neck CT scans that clearly visualized the shoulder joint region without any evident pathology. Cases with radiological evidence or documented clinical history of glenoid fractures, shoulder arthritis, tumors involving the shoulder region, glenohumeral instability, prior shoulder surgery, or significant deformity were excluded based on radiology reports and available medical records. All imaging records were de-identified before analysis, and no personally identifiable information was accessed.

**Figure 1:**
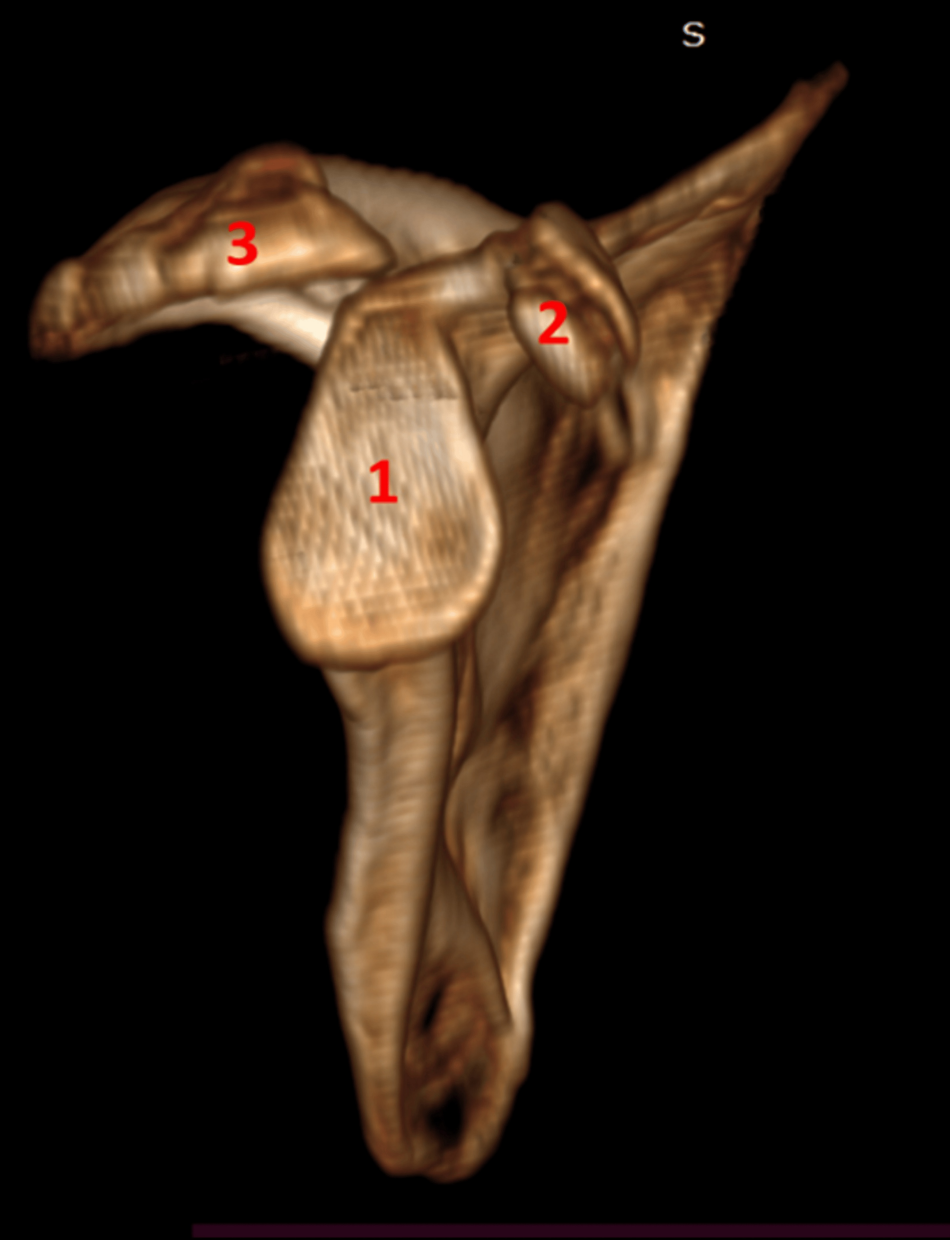
3D CT scan of the right scapula, lateral view The image illustrates the glenoid fossa (1), coracoid Process (2), and acromion process (3) CT: computed tomography

All scans were performed using a Philips Brilliance 128-slice CT scanner. The scanning area extended from the base of the skull to the upper thorax. The imaging parameters included a pitch of 1.078, acquisition of 64 × 0.625 mm collimation, rotation time of 0.75 seconds, and tube settings of 120 kV at 200 mAs. Images were reconstructed using a 0.9 mm slice thickness for multiplanar reconstruction (MPR), maximum intensity projection (MIP), and volume rendering techniques. Image analysis was performed using the Philips Extended Brilliance Workspace. The glenoid fossa was assessed (Figure 1) using electronic calipers at a standardized location [15].

**Figure 2:**
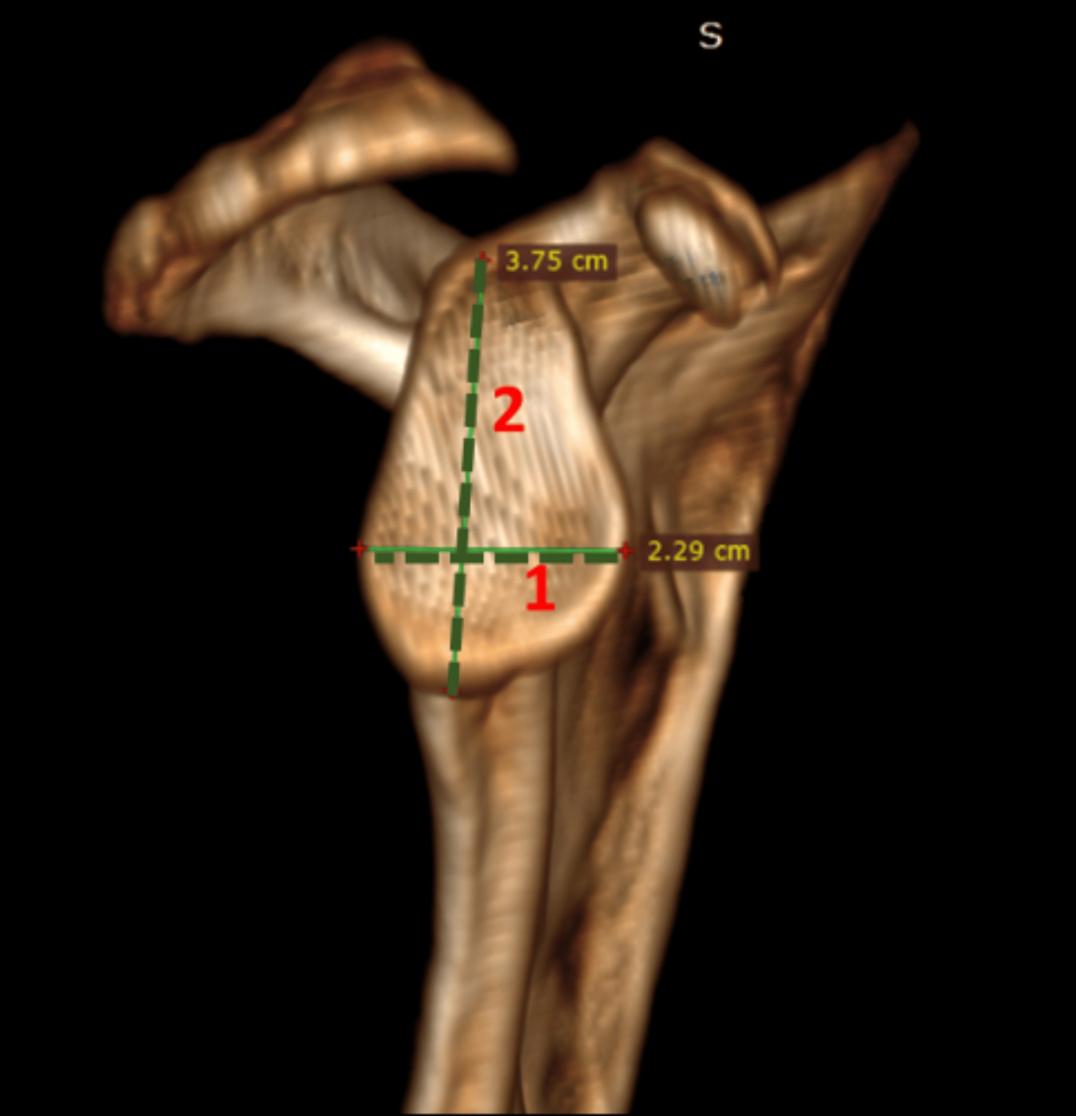
3D CT lateral view of the right scapula showing measurement of the glenoid fossa 1: maximum anteroposterior diameter of the glenoid (glenoid width); 2: maximum supero-inferior diameter (glenoid height) CT: computed tomography

Glenoid width was defined as the maximum distance between the anterior and posterior margins of the glenoid, measured in the lateral view of the reconstructed 3D CT scan [16]. Glenoid height was measured from the most superior to the most inferior point of the glenoid fossa in the same lateral view (Figure 2) [17]. GCI was used to assess glenoid morphology, calculated as the ratio of glenoid width to glenoid height C 100, as originally described by Churchill et al. [18]. GCI is an open-access morphometric parameter.

**Figure 3:**
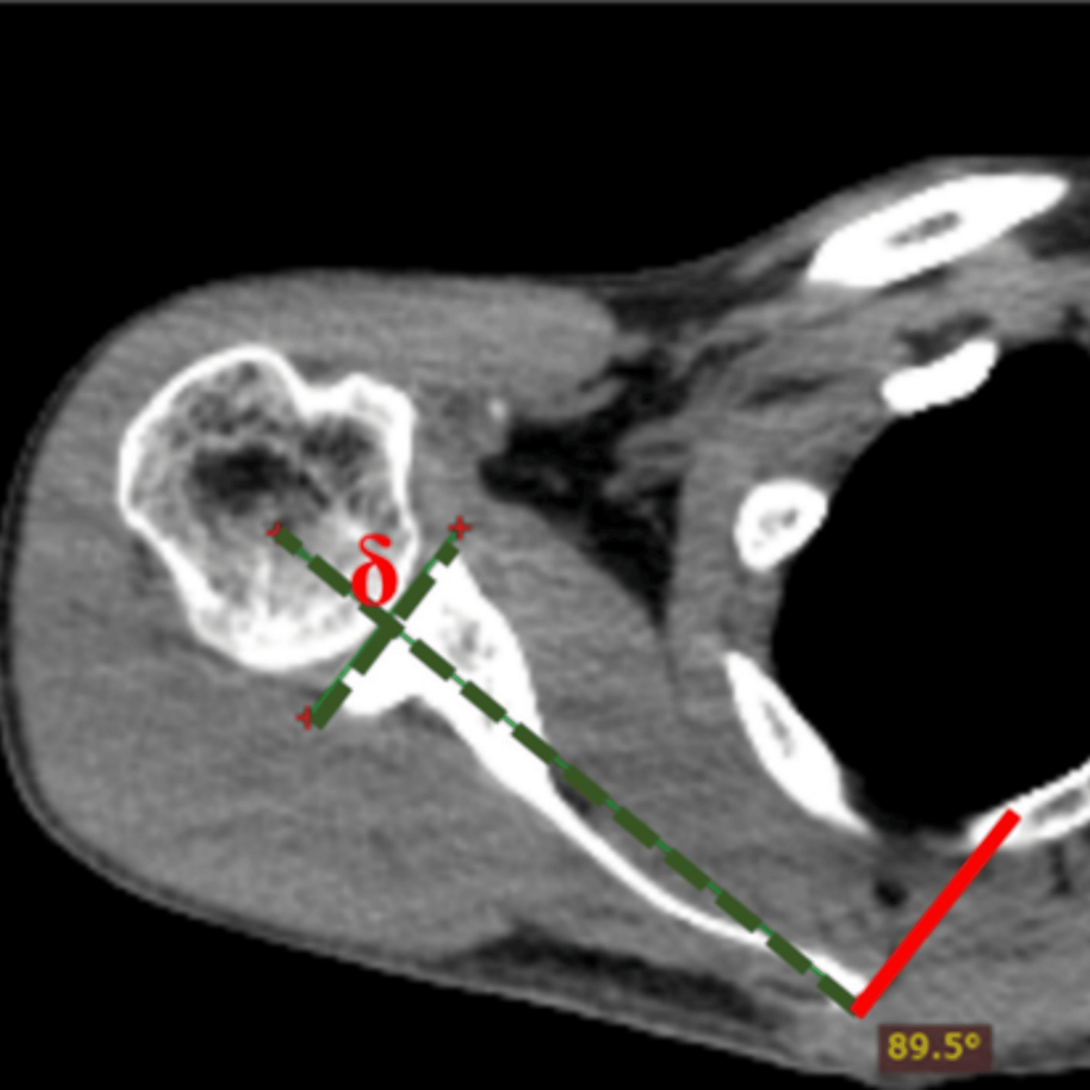
2D axial CT image of the right scapula showing glenoid version angle measurement The first line is drawn from the mid-point of the glenoid with the vertebral border of the scapula, and the second line is drawn from the anterior and posterior margins of the glenoid; the angle subtended by these two lines was marked angle δ CT: computed tomography

The glenoid version was measured in the axial view of the 2D multiplanar CT images. The first line was drawn connecting the mid-point of the glenoid to the vertebral border of the scapula. A second line was drawn between the anterior and posterior margins of the glenoid. The angle (δ) formed between these two lines was used to calculate the version angle (β) [19]. The glenoid fossa was categorized as retroverted (β < 0) or anteverted (β > 0), with the version angle computed using the formula: β = δ − 90° [17]. This measurement was taken at the mid-glenoid level, specifically four slices inferior to the coracoid process (Figure 3). The distribution of subjects by age group was as follows: 30-39 years (n = 15), 40-49 years (n = 25), 50-59 years (n = 19), 60-69 years (n = 12), and >70 years (n = 4).

Statistical analysis was performed using SPSS Statistics Software, version 26.0 (IBM Corp., Armonk, NY). Continuous variables, including glenoid width, height, version, and GCI, were expressed as mean ± standard deviation (SD), while categorical variables were presented as frequencies and percentages. Data distribution was assessed for normality before analysis. Paired t-test was used for comparison between right and left sides of the same individual, whereas the independent sample t- test was used for comparison between males and females. Pearson’s chi-square test was applied to evaluate the association between glenoid version categories, laterality, and sex. A p-value < 0.05 was considered statistically significant.

## Results

Summary of glenoid fossa dimensions and version

The mean glenoid width and height on the left side were 23.04 ± 2.75 mm (range: 18.30 - 29.80 mm) and 38.35 ± 4.26 mm (range: 30.30 to 48.40 mm), respectively, while on the right side, they were 23.10 ± 2.45 mm (range: 18.10 - 28.10 mm) and 38.14 ± 3.86 mm (range: 31.60 - 49.10 mm), respectively. Paired t-test analysis showed no statistically significant difference between the sides in mean glenoid width (t = -0.31, p = 0.757) and height (t = 0.70, p = 0.487) (Table 1). The mean left glenoid version was 2.41 ± 4.32 degrees (range: -6.10 retroversion to 13.60 anteversion), while the mean right glenoid version was 0.09 ± 4.55 degrees (range: -8.50 retroversion to 11.70 anteversion). This difference was statistically significant, indicating a significant variation in glenoid version between the sides (t = 5.17, p < 0.001) (Table 1).

**Table 1:** Comparison of the dimensions of the glenoid fossa between the left and right sides SD: standard deviation

Dimension	Left, mean ± SD	Min	Max	Right, mean ± SD	Min	Max	t-value	p-value
Glenoid width (mm)	23.04 ± 2.75	18.30	29.80	23.10 ± 2.45	18.10	28.10	–0.31	0.757
Glenoid height (mm)	38.35 ± 4.26	30.30	48.40	38.14 ± 3.86	31.60	49.10	0.70	0.487
Glenoid version (°)	2.41 ± 4.32	–6.10	13.60	0.09 ± 4.55	–8.50	11.70	5.17	<0.001

Age-wise distribution

The mean age of the study subjects was 49.12 ± 11.61 years. The largest number of subjects belonged to the age group of 40 to 49 years (25; 33.3%), followed by the age group of 50 to 59 years (19; 25.3%). The other age groups (30 to 39 years, 60 to 69 years, and 70 years or older) were represented in proportions of 20% (15), 16% (12), and 5.3% (4), respectively. The statistical analysis revealed that the mean left glenoid width was highest in the age group 70 years or older (24.20 ± 3.34) and lowest in the age group 40 to 49 years (22.75 ± 2.57); however, no significant difference was found (F = 0.41, p = 0.800). Similarly, the mean right glenoid width was highest in the age group 60 to 69 years (23.65 ± 2.37) and lowest in the age group 50 to 59 years (22.85 ± 2.28), without a statistically significant difference (F = 0.23, p = 0.919). The mean left glenoid height was highest in the age group 60 to 69 years (40.70 ± 4.00) and lowest in the age group 70 years or older (36.30 ± 4.50), with no significant difference found (F = 1.76, p = 0.148).

**Figure 4:**
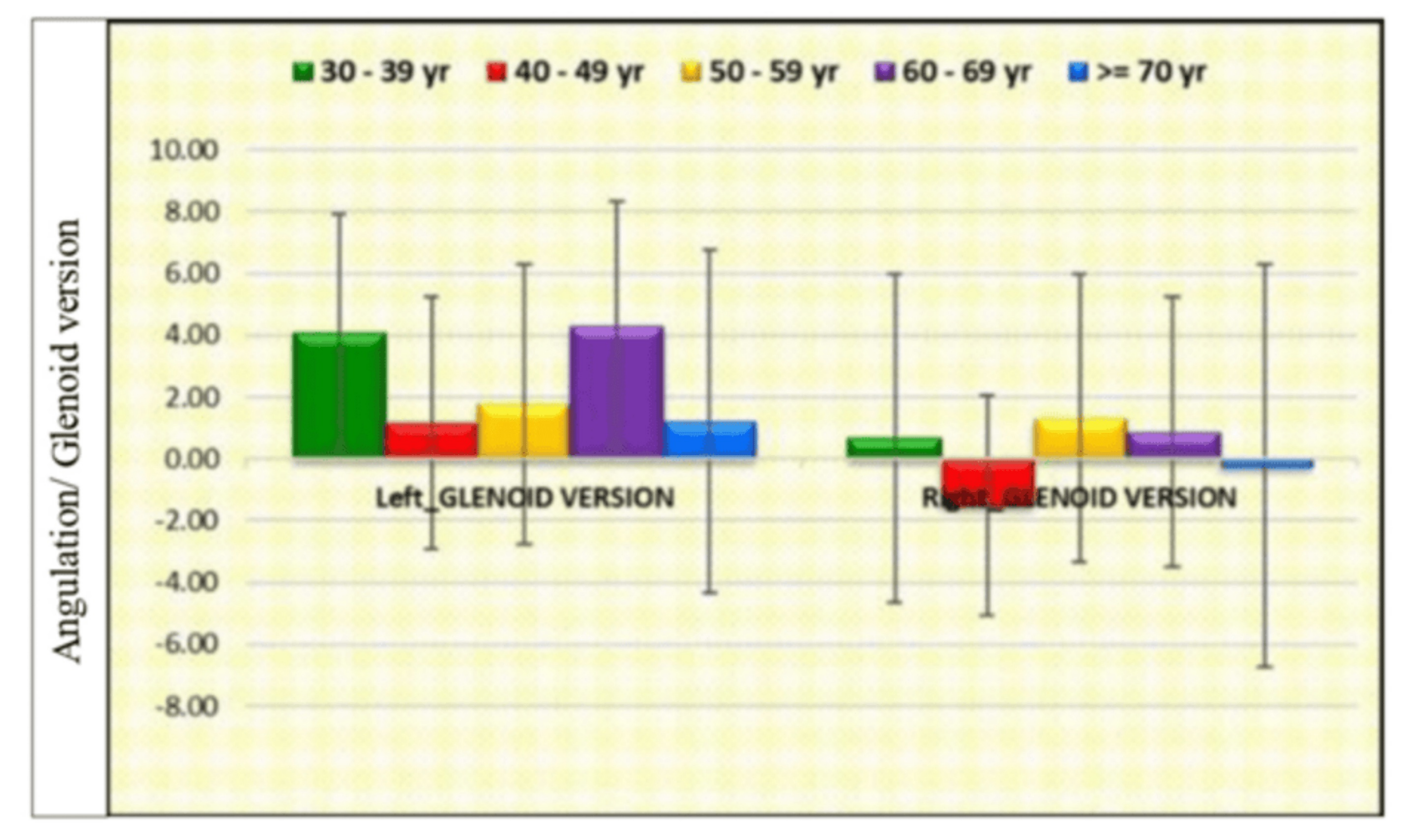
Clustered bar diagrams showing the comparison of glenoid version dimensions across different age groups The figure illustrates the comparison of glenoid version dimensions across different age groups. The distribution of subjects by age group was as follows: 30 - 39 years (n = 15), 40 - 49 years (n = 25), 50 - 59 years (n = 19), 60-69 years (n = 12), and >70 years (n = 4)

Similarly, right glenoid height was highest in the age group 60 to 69 years (40.56 ± 4.17) and lowest in the age group 40 to 49 years (36.84 ± 3.36), with no significant difference found among the age groups (F = 2.13, p = 0.087) (Figure 4, Table 2). The mean left glenoid version was highest in the age group 60 to 69 years (4.33 ± 4.02 degrees) and lowest in the age group 70 years or older (1.23 ± 5.56 degrees anteverted), with no significant difference between age groups (F = 1.95, p = 0.111). The mean right glenoid version showed maximum retroversion in the age group 40 to 49 years (-1.50 ± 3.55 degrees) and minimum retroversion in the age group 70 years or older (-0.23 ± 6.51 degrees) (Figure 4, Table 2), with no statistically significant difference observed (F = 1.28, p = 0.286).

**Table 2:** Comparison of the dimensions of the glenoid fossa with age SD: standard deviation

Dimension	30-39 years, mean ± SD	40-49 years, mean ± SD	50-59 years, mean ± SD	60-69 years, mean ± SD	≥70 years, mean ± SD	F-value	p-value
Left glenoid width (mm)	23.13 ± 2.89	22.75 ± 2.57	22.75 ± 2.59	23.59 ± 3.28	24.20 ± 3.34	0.41	0.800
Right glenoid width (mm)	23.19 ± 2.61	22.94 ± 2.58	22.85 ± 2.28	23.65 ± 2.37	23.35 ± 3.09	0.23	0.919
Left glenoid height (mm)	37.29 ± 4.01	37.64 ± 3.62	39.07 ± 4.98	40.70 ± 4.00	36.30 ± 4.50	1.76	0.148
Right glenoid height (mm)	38.02 ± 3.44	36.84 ± 3.36	38.58 ± 4.07	40.56 ± 4.17	37.25 ± 4.33	2.13	0.087
Left glenoid version (°)	4.09 ± 3.80	1.16 ± 4.09	1.77 ± 4.52	4.33 ± 4.02	1.23 ± 5.56	1.95	0.111
Right glenoid version (°)	0.66 ± 5.32	-1.50 ± 3.55	1.31 ± 4.66	0.88 ± 4.36	–0.23 ± 6.51	1.28	0.286

Sex-based differences in glenoid dimensions and version

**Figure 5:**
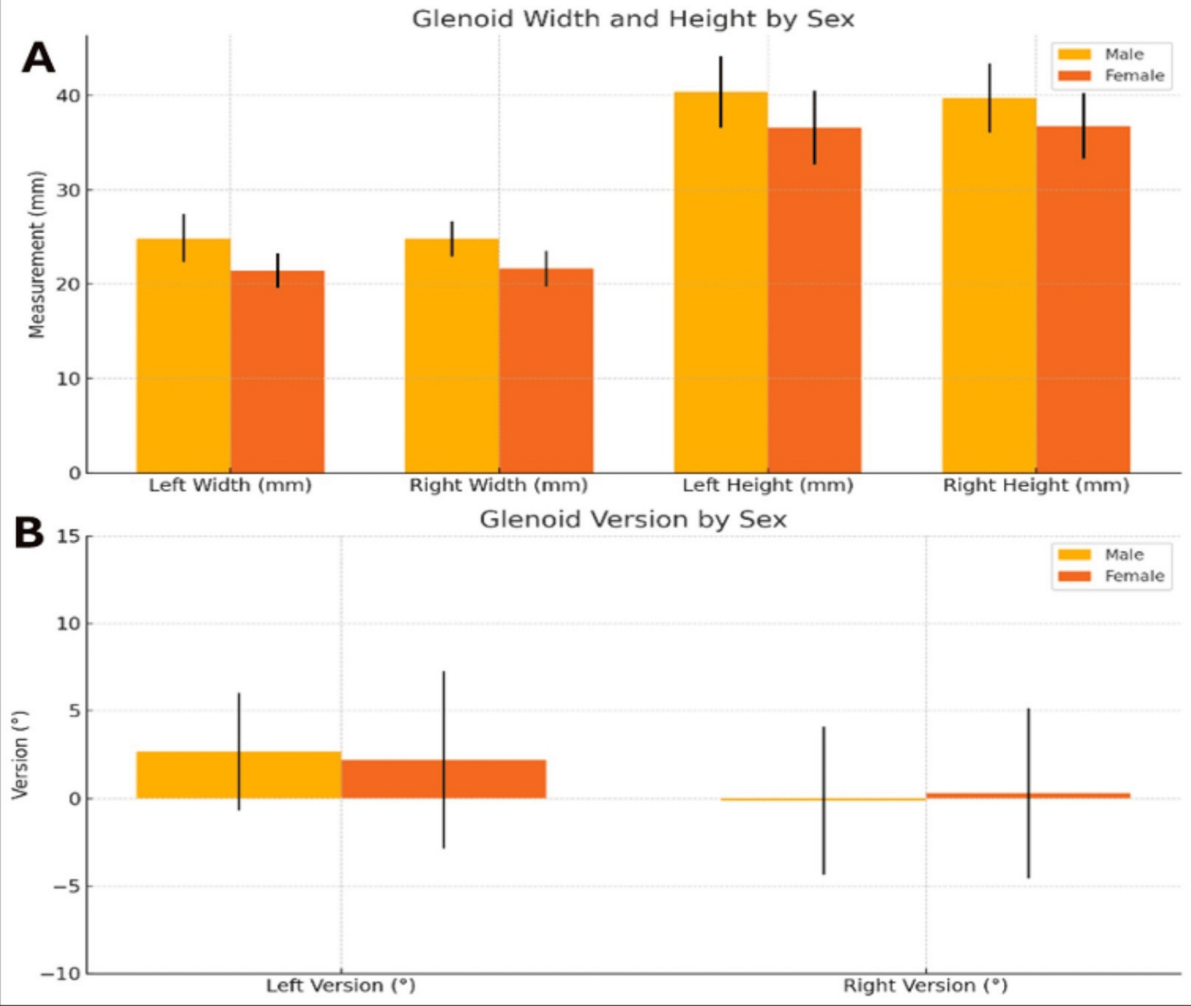
Clustered bar diagrams showing the comparison of dimensions of the glenoid fossa (A) and version (B) with gender

The mean left glenoid width of males and females was 24.86 ± 2.54 and 21.45 ± 1.78, respectively, showing a significant difference (t = 6.80, p < 0.001). Similarly, the mean right glenoid width in males and females was 24.79 ± 1.88 and 21.63 ± 1.88, respectively, indicating a significant difference (t = 7.26, p < 0.001). The mean left glenoid height of males and females was 40.36 ± 3.78 and 36.59 ± 3.90, respectively, with a significant difference (t = 4.42, p < 0.001). The mean right glenoid height of males and females was 39.72 ± 3.67 and 36.75 ± 3.51, respectively, also showing a significant difference (t = 3.57, p < 0.001) (Figure 5A, Table 3). The mean left glenoid version of males was 2.66 ± 3.36 (range: -4.90 to 9.30 degrees), while that of females was 2.20 ± 5.05 (range: -6.10 to 13.60 degrees), with no significant difference (t = 0.46, p = 0.645). The mean right glenoid version of males was -0.14 ± 4.23 (range: -8.20 to 7.70 degrees), while that of females was 0.29 ± 4.85 (range: -8.50 to 11.70 degrees) (Figure 5B, Table 3), with no significant difference (t = 0.40, p = 0.688).

**Table 3:** Comparison of the dimensions of the glenoid fossa with sex SD: standard deviation

Dimensions of the glenoid fossa	Male		Female		Unpaired t-test	
	Mean	SD	Mean	SD	t-value	p-value
Left glenoid width (mm)	24.86	2.54	21.45	1.78	6.80	0.000
Right glenoid width (mm)	24.79	1.88	21.63	1.88	7.26	0.000
Left glenoid height (mm)	40.36	3.78	36.59	3.90	4.24	0.000
Right glenoid height (mm)	39.72	3.67	36.75	3.51	3.57	0.001
Left glenoid version (°)	2.66	3.36	2.20	2.20	0.46	0.645
Right glenoid version (°)	-0.14	4.23	0.29	4.85	0.40	0.688

Glenoid retroversion on the left side was observed in seven (9.3%) males and 19 (25.3%) females, whereas on the right side, it was noted in 17 (22.7%) males and 24 (32%) females. Overall, glenoid retroversion was more frequent on the right side (41 joints; 54.7%) compared with the left side (26 joints; 34.7%). In contrast, glenoid anteversion was more commonly observed on the left side (49 joints; 65.3%) than on the right side (34 joints; 37.3%). Glenoid anteversion on the left side was present in 28 (37.3%) males and 21 (28.0%) females, while on the right side, it was observed in 18 (24.0%) males and 16 (21.3%) females. A significant association of glenoid version was found with laterality (chi-squared (1) = 6.07, p = 0.014). A statistically significant association of glenoid version was also found with sex (chi-squared (1) = 6.23, p = 0.012) on the left side, but not on the right side (chi-squared (1) = 0.98, p = 0.321) (Table 4).

**Table 4:** Comparison of glenoid versions with laterality and sex

Glenoid version		Left		Right		Left vs. right
		N	%	N	%	
Glenoid retroversion	Male	7	9.3	17	22.7	Chi-squared (1) = 6.07, p= 0.014
	Female	19	25.3	24	32.0	
	Total	26	34.7	41	54.7	
Glenoid anteversion	Male	28	37.3	18	24.0	
	Female	21	28.0	16	21.3	
	Total	49	65.3	34	45.33	
Male vs. female		Chi-squared (1) = 6.23, p = 0.012		Chi-squared (1) = 0.98, p = 0.321		Total

Glenoid cavity index (GCI)

The mean GCI on the left was 59.18 ± 7.3% in females and 62.35 ± 10.1% in males, with no statistically significant difference (t = 1.88, p = 0.064). On the right, values were 59.24 ± 6.4% and 63.02 ± 8.2% in females and males, respectively, with a statistically significant difference (t = 2.48, p = 0.0159). Overall, the mean GCI of females and males was 59.21 ± 6.8% and 62.68 ± 9.1%, respectively, with a statistically significant p-value of 0.0102 (t = 2.64) (Table 5). No statistically significant association was observed between laterality and GCI (t = 0.86, p = 0.389). These findings indicate sexual dimorphism in glenoid morphology, which may have implications for prosthesis design and preoperative shoulder surgical planning.

**Table 5:** Comparison of GCI with laterality and sex GCI: glenoid cavity index; SD: standard deviation

Comparison	Female GCI, (%), mean ± SD	Male GCI, (%), mean ± SD	t-value	p-value
Left side	59.18 ± 7.3	62.35 ± 10.1	1.88	0.064
Right side	59.24 ± 6.4	63.02 ± 8.2	2.48	0.0159
Overall (male vs. female)	59.21 ± 6.8	62.68 ± 9.1	2.64	0.0102
Laterality (left vs. right)	—	—	0.86	0.389

## Discussion

The understanding of glenoid fossa anatomy is essential in modern shoulder surgery, particularly in TSA, RTSA, and reconstructive procedures addressing instability or bone loss. The glenoid exhibits substantial anatomical variability across populations, and glenoid component malposition, inadequate bone coverage, or uncorrected version abnormalities can lead to implant loosening and early failure. Accurate morphometric characterization is essential for optimizing implant positioning and minimizing postoperative complications. The present study provides important insights into glenoid morphometry by demonstrating significant sexual dimorphism in glenoid height, width, and GCI, whereas age-related differences were not statistically significant.

Males exhibited greater glenoid height, width, and overall GCI values compared to females. The observed glenoid height and width measurements were comparable with the findings of Mathew et al. (2017) and Pinak et al. (2015), both of whom reported larger glenoid dimensions in males. Similarly, the sex-based variation in GCI observed in the present study was consistent with previous morphometric studies by Dhindsa et al. (2014), Polguj et al. (2011), and Tanaka et al. (2023). In contrast, Piponov et al. (2016) documented relatively lower mean glenoid dimensions, possibly reflecting population-specific anatomical variation. Additionally, glenoid version in the present study remained within previously reported physiological ranges, with minor side-related variation but no significant sex-related differences.

Variations in glenoid height and width by gender and age

The study observations reported by Pinak et al. (2015) [20] documented a mean glenoid height of approximately 38 mm with a noticeable gender difference, and Piponov et al. (2016) [21] reported a mean height of 31.7 ± 3.7 mm with measurable sex-based variation. Similarly, Mathew et al. (2017) [17] reported mean heights of 39.5 ± 3.5 mm in males and 34.8 ± 2.2 mm in females. In the present study, glenoid height demonstrated significant sexual dimorphism, with higher mean values observed in males on both sides (left: 40.36 ± 3.78 mm; right: 39.72 ± 3.67 mm) compared to females (left: 36.59 ± 3.90 mm; right: 36.75 ± 3.51 mm), with strong statistical significance (p < 0.001), although no age-related differences were significant. 

In the present study, the mean glenoid width in males was 24.86 ± 2.54 mm (left) and 24.79 ± 1.88 mm (right), while in females it was 21.45 ± 1.78 mm (left) and 21.63 ± 1.88 mm (right). These findings were consistent with previous studies by Pinak et al. (2015), who reported a glenoid width of 26.8 mm with a 4.2 mm gender difference [20], and Mathew et al. (2017), who reported 27.8 ± 3.3 mm [17]. However, Piponov et al. (2016) [21] stated that these parameters correlate not only with patient gender but also with ethnicity, which may reflect population-specific skeletal patterns. The absolute width values in the present study were slightly lower than those reported in Western cohorts, highlighting regional variation and the importance of developing localized morphometric databases for implant design.

Regarding age, Knapik et al. (2018) reported that the highest and lowest mean glenoid widths were 29.4 ± 2.9 mm in the ≥ 70-year age group and 27.9 ± 2.7 mm in the 30-39-year age group, respectively, indicating an increase with age [22]. In the present study, comparison of age with glenoid width revealed that the maximum mean glenoid width was 24.20 ± 3.34 mm (left) and 23.65 ± 2.37 mm (right) in the ≥ 70-year age group, while the minimum was 22.75 ± 2.57 mm (left) and 22.85 ± 2.28 mm (right) in the 40-49-year age group. This discrepancy may reflect differences in sample size and also suggests that age-related remodeling may influence glenoid dimensions.

Glenoid cavity index

The GCI, reflecting glenoid shape rather than size, was notably lower in the present cohort compared to prior studies. Tankala M. et al. (2023) [23] reported mean GCIs of 68.84 ± 7.64% (left) and 68.44 ± 7.98% (right), while Polguj et al. (2011) [8] reported 72.35 ± 5.55%. Dhindsa et al. (2014) [7] revealed mean GCIs of 68.59 ± 4.36% (left) and 70.37 ± 4.08% (right), whereas Hassanein et al. (2015) [24] noted higher mean values of 76.71 ± 8.37% (left) and 73.67 ± 9.08% (right) in dry human scapulae. In contrast, the present study showed GCIs with a gender difference: 59.18 ± 7.3% (female) and 62.35 ± 10.1% (male) on the left (p = 0.064), and 59.24 ± 6.4% (female) and 63.02 ± 8.2% (male) on the right (p = 0.0159). A lower GCI indicates a relatively taller and narrower glenoid morphology. Biomechanically, such a configuration may reduce transverse baseplate coverage during RTSA and increase the risk of peripheral stress concentration [7,24].

Glenoid version

Glenoid version demonstrated substantial variation. Piponov et al. (2016) [21] reported a mean glenoid version of 2.65 ± 9.01° in females and -1.65 ± 9° in males, while Mathew et al. (2017) [17] noted that the mean glenoid version ranged from -13.5° to 4.5° in human bones and -10° to 10° on CT scans. In the present study, the mean glenoid version in males was 2.66 ± 3.36° (left) and -0.14 ± 4.23° (right), while in females it was 2.20 ± 2.20° (left) and 0.29 ± 4.85° (right). The ranges were -6.10° to 13.60° on the left side and -8.50° to 11.70° on the right side, nearly in concordance with the findings of Mathew et al. (2017) [17]. A significant difference (p < 0.001) was observed between the left and right glenoid version values, possibly due to limb dominance and asymmetric loading. However, no significant gender-based difference was found.

The glenoid version was highest in the 60-69-year age group and lowest in the ≥ 70-year age group, but no significant differences were found among the age groups. Clinically, unrecognized side-specific retroversion or anteversion may predispose the shoulder to eccentric loading and posterior instability. Persistent retroversion beyond physiological thresholds has been associated with an increased risk of glenoid component loosening. These results reinforce previous findings and highlight that variability in glenoid version is influenced primarily by laterality rather than by gender or age.

The present study has certain limitations. It was a retrospective, single-center study with a relatively small sample size, which may limit generalizability. Additionally, measurements were obtained from head and neck CT scans that incidentally included the shoulder region, rather than from dedicated shoulder imaging, potentially introducing selection bias. Multicentric studies with larger sample sizes could provide more robust conclusions.

## Conclusions

The present CT scan-based study establishes normative data for glenoid fossa dimensions, which may serve as a valuable reference for surgeons and radiologists in distinguishing normal anatomical variations from pathological conditions in clinical practice. The findings indicate that sex-based differences were observed in glenoid dimensions and GCI, while glenoid version showed a significant association with laterality (p = 0.014). The present study also demonstrated minor side-related variation in glenoid version, consistent with previous studies, although the magnitude of this difference is likely within physiological limits in the absence of significant pathology. These observations may be particularly relevant during radiological assessment and preoperative evaluation of shoulder disorders.
